# Competency-based education is beneficial for professional development

**DOI:** 10.1007/s40037-015-0232-6

**Published:** 2015-11-09

**Authors:** Cees P.M. van der Vleuten

**Affiliations:** Department of Educational Development and Research, Faculty of Health, Medicine and Life Sciences, Maastricht University, PO Box 616, 6200 MD Maastricht, The Netherlands

## Essentials

- Competency-based education promotes relevant skills for medical training.
- Competency-based education promotes better health care.
- Competency-based education promotes better curriculum governance.

Competency-based education is a logical next step in professionalizing education. A competency is the integration of knowledge, skills and attitudes to successfully engage with a professional task. Competency-based education is when a curriculum or training programme provides holistic tasks to practise these competencies. We come from an era in which education was defined by its input. For example, we need at least X hours of anatomy and Y hours of physiology in the curriculum. In an input model curriculum, there is a constant hidden, and sometimes overt, fight over hours of curriculum representation. This had led to many overloaded curricula around the world. Educationalists then started pushing for the formulation of objectives to better structure an education programme. Competencies are in essence an alternative to objectives, but then focusing on the end objectives of a training programme in terms of what learners should be able to do. Once you know what the end product should be able to do, you can systematically look at your training programme (including the assessment programme) and deliberately cater your education to these end objectives. Many countries in the world have developed consensus on these competencies, in so-called competency frameworks. The most well-known are the frameworks from the Accreditation Council of Graduate Medical Education from the US [1], the CanMEDS framework from Canada [2] and Good Medical Practice from the UK [3]. There are many other countries in the world that have developed their own competency framework acknowledging their own local needs for training programmes in their competency definitions. Unlike my opponent [4], I am in favour of having these competency frameworks for 2 main reasons.

## Embracing competencies will have positive health care consequences

All competency frameworks have been developed with considerable stakeholder input. In addition to relevant medical input, societal parties such as assurance companies, employers, regulators and patients were included. All I am saying is that these frameworks have not been developed overnight. They have a solid foundation, firmly rooted in what society wants our doctors to be. It is then striking to note how similar these competency frameworks are. The competencies have different names in different frameworks but when one starts looking at the descriptions underneath they do have incredible similarity. This similarity and their robust development assure me we have consensus on what the purpose should be of medical training, either undergraduate or postgraduate.

Where does this consensus point us? Very clearly it is to the emphasis of skills beyond the knowledge domain. I guess our society kind of assumes that our doctors are knowledgeable, but society wants more. They want compassionate and communicative doctors who act professionally [5]. Doctors need to work in teams, need to collaborate with other professions, need to engage in life-long learning activities, need to be transformative to their working environment [6]. These skills have been given different names (soft skills, employability skills, 21st century skills, generic skills). No skill is ever generic and rather contextually bound, but they are generic to the domain. Therefore I prefer to call them domain-independent skills. These skills are not unique to the domain of medicine and are relevant for any profession.

Domain-independent skills are not only important because our stakeholders have identified them. There is solid research showing that both poor and good functioning on the labour market is associated to these skills. There are the famous Papadakis studies showing how behavioural lapses in a clinical work environment were preceded by professionalism lapses in the training programme [7]. I myself have been involved in a study looking at hospital complaints and elements of communication and professionalism were the common ones [8]. Other studies have demonstrated that success in the labour market often involves people who have excellent domain-independent skills [9, 10].

My first argument is that competency-based education has called our attention to skills that up until now we have always treated implicitly. If we address these skills properly, we will serve the community and the patient better and our health care will be improved.

## Embracing competencies for learning has formidable educational consequences

I have been part of many curriculum revisions in the past. It was striking how easy it was to discuss issues of knowledge that learners need to master. However, re-orientating the discussion on domain-independent skills was extremely difficult. There is a natural inclination by teachers to exclusively focus on the cognitive component, ignoring other competencies. If we seriously adopt a competency framework, then it has serious consequences for developing a curriculum.

First, all domain-independent competencies are complex behaviours. Complex behaviours are not learned in a course of a couple of weeks followed by an exam and once passed you master that competency. One cannot take a three-week training programme in communication skills with an OSCE at the end and emerge as a good communicator. These complex skills need longitudinal development, longitudinal nurturing and monitoring. These competencies typically form the domains crossing the whole curriculum. Training programmes in medicine have great difficulty with longitudinal strands in a curriculum. Our most common notion of learning is behaviourist: a learner is instructed and the passing of the ensuing exam is proof of (eternal) competence. Good learning is much more developmental and pays attention to transfer (from knowing to doing, from context to context). That is why we now have integrated curricula, early skills training, spiral curricula, active learning methods, and mentoring programmes. Competencies form the tools, the language and the leverage to discuss curriculum content. What is the role of a certain course in relation to the final product? Why is what you offer relevant? How could you make it more relevant? These questions have a profound effect on the nature of a training programme. Implicitly they require good governance on the curriculum. Individual teachers will never have the complete overview. Good governance of curricula is an innovation of a formidable nature in itself. Competencies provide the tools and a language.

Another consequence of embracing competencies is an educational one and one that deeply challenges us on how to exactly train these competencies. I have seen medical students being trained on communication in a lecture theatre. I have seen a course on ethics given by lectures only. How can this be more than a written course on karate? Complex behaviours are NOT learned in a classroom. They are learned by doing, by experiencing, by getting feedback, by new experimentation, etc. In recent studies on communication [11, 12], residents learned communication by first using ‘learned’ and somewhat artificial techniques in actual practice, then with ample feedback on authentic clinical actions (video assessment, peer feedback, supervisor interactions); under safe conditions, communication behaviours become personalized and internalized. Finally, communication behaviours become part of the personal clinical repertoire and can be used flexibly, all depending on the context and the purpose of the encounter. This challenges us to look at our training programmes educationally? How much feedback do we give to learners? How much safety do we provide? How much of our assessment is truly authentic? If we truly embrace competency-based education, there is still a long way to go.

## Some disclaimers

My heartfelt plea in favour competency-based education has not clouded my view on the world. Competency-based education is not a hammer in a world of nails. A couple of disclaimers are important to make.

The reader should not confuse my plea for the importance of domain-independent skills with the unimportance of knowledge. Knowledge is at the heart of every domain. All the research on expertise development in medicine has clearly shown how important knowledge is for being able to function as an expert [13]. This is undisputed. Will the attention to domain-independent skills compromise the knowledge? The answer seems to be no. There is upcoming research showing that paying attention to generic skills helps to better equip learners, *not* at the cost of knowledge [14–16].

I am very much aware that competency-based learning may confuse teachers and clinicians. Consensus definitions of what health advocacy is or professionalism do not exist. I applaud the recent notion of Entrustable Professional Activities (EPAs) [17]. Clinicians can easily come up with critical professional activities to be entrusted to their learners. With EPAs understanding competencies becomes easier. An obstetrician will know exactly with whom to collaborate and communicate in a normal child delivery situation. The recent development of training programmes explicating EPAs and milestones of development is a good one [18]. The downside may be in its use. If we use them too strictly, train them too strictly on detailed lists of mandatory performance, the nature of learning may be trivialized. We might be ending with a modern form of behaviourism. The value of competency-based education, including EPAs and milestones, is that we make explicit what we expect from our learners in a language that the professionals in the domain understand naturally. Like anything else, we can overdo this and harm the learning. What is important on paper should become a personalized and flexible clinical repertoire associated with good health care outcomes. The art of medicine will remain intact. Competency-based education is a way to make the art a little bit more explicit.

## Epilogue

Embracing competency-based education in a proper way will have beneficial effects on health care and on teaching and learning. Truly embracing competency-based education provides us with very many challenges. There is one relief though. There is a lawful relationship in education stating that whatever we pay attention to will grow. Education matters!
